# Screening of natural compounds library for protection against endoplasmic reticulum stress in corneal endothelial cells

**DOI:** 10.3389/fopht.2026.1945775

**Published:** 2026-09-24

**Authors:** Stephanie Lee, Anisha Kasi, Minwoo Kwon, Stefan Y. Kim, Saba Qureshi, Varun Kumar

**Affiliations:** 1 Eye and Vision Research Institute, Department of Ophthalmology, Icahn School of Medicine at Mount Sinai, New York, NY, United States; 2 Department of Pharmacological Sciences, Icahn School of Medicine at Mount Sinai, New York, NY, United States

**Keywords:** apoptosis, corneal endothelial cells, drug screening, endoplasmic reticulum stress, Fuchs endothelial corneal dystrophy, natural products, unfolded protein response, veratrine

## Abstract

**Purpose:**

Endoplasmic reticulum (ER) stress-mediated cell death contributes to many ocular diseases, including Fuchs corneal endothelial dystrophy (FECD). Natural compounds possess significant pharmacological activities necessary for vital cell signaling pathways and are considered protective in many ocular diseases such as diabetes, retinopathy, and glaucoma. However, there are limited studies examining the role of natural compounds in reducing sustained ER stress for improving corneal endothelial cell (CEnCs) survival. The purpose of this study is to identify readily available natural compounds to improve the viability of CEnCs under ER stress.

**Methods:**

Human corneal endothelial cells (21T-HCEnC) were cultured and seeded in 384-well plates, pre-treated with 502 compounds from a natural product library (Enzo Screen-Well Natural Product library), and then subjected to ER stressor tunicamycin (1µg/mL) for 24 hours. Cell viability was measured using the Cell-Titer-Glo assay, and 74 compounds improved viability past the set threshold (≥ 30% increase in viability) and were selected for further dose-response testing. Further dose-response testing was performed using untreated F35T corneal endothelial cells, plated onto 384-well plates, and treated with a range of 6 log doses of each compound, from 10 pg/mL up to 1 μg/mL. After 24 hours of treatment, cell viability was measured again using the Cell-Titer Glo assay. Of the 4 natural compounds that improved viability past the set threshold at the lowest dose tested (0.00001 µg/mL), veratrine was analyzed further via western blotting, ranging from 10 pg/mL to 0.01 μg/mL. Apoptotic and unfolded protein response markers were also measured via western blotting.

**Results:**

Of 502 compounds each administered at 0.01 µg/mL, we identified 74 compounds exhibiting 30% or more protection of 21T HCEnC cell viability in response to tunicamycin-induced stress. Of the 74 compounds that underwent further dose-response testing, 4 compounds improved cell viability by 30% or more at the lowest dose of compound tested compared to viability of F35T cells at baseline. Cevadine, or veratrine, was chosen for further analysis due to its function as a sodium channel agonist and its interaction with Na^+^/K^+^ ATPase channels. Pretreatment with veratrine 0.01 µg/mL, along with tunicamycin, downregulated expression levels of cleaved caspase 9, PARP, cytochrome C, Bax, and cleaved caspase 3. Western blots of FECD cell line under tunicamycin-induced ER stress, pretreated with veratrine 0.01 µg/mL, also showed reduced markers from all three ER unfolded protein response pathways.

**Conclusions:**

This study provided insight into the role of natural compounds in regulating ER stress-mediated CEnC death associated with many corneal endothelial-associated diseases. Veratrine was found to have a protective effect on ER-stressed CEnCs, as indicated by reduced apoptotic signals and ER stress markers following veratrine treatment, suggesting a possible mechanism of action via mediating ER stress signals. Future studies are needed to clarify the mechanism and utility of veratrine in FECD.

## 1 Introduction

Fuchs corneal endothelial dystrophy (FECD) is a progressive degenerative disease of the corneal endothelium, affecting roughly 300 million people (1). FECD is defined by the loss of corneal endothelial cells (CEnC) and the formation of extracellular deposits called guttae, contributing to corneal edema and vision loss (2). Currently, there is no cure for this dystrophy; thus, it remains the leading cause of corneal transplants worldwide (3). Although endothelial keratoplasty has significantly improved visual outcomes for patients with FECD, corneal transplantation remains the only definitive treatment for advanced disease. However, transplantation is limited by donor tissue availability, risk of graft failure, continued endothelial cell loss over time, and the need for long-term postoperative care. These limitations highlight the need for medical therapies that can preserve corneal endothelial cells and slow disease progression before irreversible damage occurs.

One major factor in FECD-induced apoptosis of corneal endothelial cells is persistent upregulation of the endoplasmic reticulum (ER) unfolded protein response (UPR) pathways. Several factors, including oxidative stress, mitochondrial dysfunction, impaired protein quality control, and genetic abnormalities, have been implicated in the development of chronic ER stress in FECD (4). While activation of the UPR initially helps restore ER homeostasis, prolonged activation can become maladaptive and ultimately promote apoptosis and progressive corneal endothelial cell loss (5). The three main UPR pathways are activating transcription factor 6 (ATF6), inositol-requiring enzyme 1α (IRE1), and protein kinase RNA-like ER kinase (PERK) – activating transcription factor 4 (ATF4) – C/EBP-homologous protein (CHOP) (PERK-ATF4-CHOP) pathways, each sensing the presence of aberrant protein folding. These pathways initiate adaptive responses by reducing protein translation, increasing molecular chaperone production, and promoting the degradation of misfolded proteins to restore ER homeostasis (5, 6). However, when ER stress is prolonged, these protective mechanisms become overwhelmed, leading to mitochondrial dysfunction, activation of apoptotic pathways, and ultimately corneal endothelial cell death (7). Past studies have observed the activation of parts of the three UPR pathways along with apoptotic markers caspase 3 (executioner caspase) and caspase 9 (initiator caspase) in FECD patient-derived corneal samples, indicating the implication of ER stress in apoptosis of CEnCs (7, 8).

Several studies have explored ER stress and the pathways of the ER unfolded protein stress response as potential therapeutic targets in ocular diseases. 4-phenylbutyrate (4-PBA), an ER chaperone that prevents the aggregation of misfolded proteins, has been studied for its effects to improve trabecular meshwork drainage in glaucoma, as well as its ability as a chaperone to recover misfolded calnexin in calnexin-mutant congenital cataracts (9, 10). Other compounds that target ER stress, including tauroursodeoxycholic acid (TUDCA) and salubrinal, have shown protective effects in experimental models of retinal degeneration, glaucoma, diabetic retinopathy, and corneal endothelial injury (11, 12). Together, these findings further support targeting ER stress as a potential therapeutic strategy for ocular disease.

Similarly, natural product compounds have also been probed for their pharmacological applications in several ocular diseases. Natural products have long been recognized as an important source of therapeutic compounds because many possess antioxidant, anti-inflammatory, anti-apoptotic, and ER stress-modulating properties (13, 14). Commonly studied nutraceuticals include carotenoids, polyphenols, vitamins and minerals, omega-3 fatty acids, and other plant-derived compounds. These agents have been investigated in age-related macular degeneration, cataract, glaucoma, diabetic retinopathy, and dry eye disease, with their potential benefits largely attributed to antioxidant, anti-inflammatory, anti-apoptotic, and neuroprotective effects (15, 16). For example, curcumin has been studied in diabetic retinopathy, saffron in age-related macular degeneration, and Ginkgo biloba in glaucoma. Other naturally derived compounds, including resveratrol, epigallocatechin gallate, quercetin, berberine, forskolin, and naringenin, have shown protective effects in experimental ocular-disease models by reducing oxidative stress, inflammation, and apoptosis (13, 15). However, the evidence supporting many of these compounds remains largely preclinical, and their translation into clinical use may be limited by variability in formulation, bioavailability, dosing, and study design (16). Despite these encouraging findings, relatively few studies have investigated whether natural compounds can protect corneal endothelial cells from ER stress.

Our group’s previous work demonstrated the implication of chronic ER stress on mitochondrial dysfunction and mitochondria-mediated apoptosis leading to CEnC degeneration in FECD (8). We also explored the potential of one previously mentioned compound, 4-PBA, and found that the ER chaperone could protect against tunicamycin-induced ER stress in a healthy corneal endothelial cell line (8). These findings suggest that targeting ER stress may be a promising therapeutic approach for FECD and raise the question of whether other naturally derived compounds may provide similar protective effects.

The purpose of this study was to screen a library of natural compounds for their ability to protect corneal endothelial cells from ER stress-induced injury. We then selected the most promising compound, veratrine, for further evaluation in an FECD-derived corneal endothelial cell line to investigate its effects on ER stress signaling and apoptosis.

## 2 Materials and methods

### 2.1 Cell culture

The immortalized human corneal endothelial cell line 21T (21T HCEnC) was generously provided by Dr. Ula Jurkunas (Harvard University, Cambridge, MA), while the immortalized Fuchs corneal endothelial cell line F35T (F35T HCEnC) was obtained from Dr. Albert Jun (Johns Hopkins University, Baltimore, MD). Both cell lines were maintained as previously described (8).

### 2.2 Screening compound library

A natural product library (SCREEN-WELL Natural Product Library, BML-2865-0100, Enzo Life Sciences, https://www.fishersci.com/shop/products/screen-well-naturalproduct/NC1670122) was used to screen against 21T corneal endothelial cells under tunicamycin-induced ER stress. Cells were plated onto 384-well plates (Corning) and pretreated with the compounds (0.01 μg/mL) from the screening library for 2 hours, then subjected to ER stressor tunicamycin (1 μg/mL) and co-incubated with both compound and stressor for 24 hours. Post-treatment, cell viability was measured using the Cell-Titer Glo assay (cat no. G7572, Promega) on a luminescent plate reader (Varioskan LUX, Thermo Fisher).

### 2.3 Dose-response screening

After selection of preliminary ‘hits’, a secondary dose-response screening procedure was performed using untreated F35T corneal endothelial cells. Similarly, cells were plated onto 384-well plates and treated with a range of 6 log doses from 10 pg/mL to 1 μg/mL. After treatment for 24 hours, cell viability was once again measured using the same Cell-Titer Glo assay and luminescent plate reader to identify the final ‘hits’.

### 2.4 Targeted analysis of veratrine

F35T cells were cultured and treated with either DMSO, tunicamycin, or tunicamycin in combination with veratrine. Four log doses of veratrine were analyzed via western blotting technique, ranging from 10 pg/mL to 0.01 μg/mL. Veratrine was applied as a pretreatment to F35T cells for 2 hours, and then tunicamycin (1 μg/mL) was added along with the veratrine treatment for 24 hours. Separate controls were generated for DMSO (0.02%) and tunicamycin (1 μg/mL). RIPA lysis buffer (cat no. 89900, Thermo Fisher) supplemented with protease and phosphatase inhibitors (cat no. 78440, Thermo Fisher) was used to collect the cells. Protein concentration of each sample was determined with the Pierce BCA Assay kit (cat no. 23227, Thermo Scientific). The samples were processed using LDS sample buffer and reducing agent, then subjected to electrophoresis through 4-12% SDS-PAGE gels, and then transferred to PVDF membranes. Membranes were blocked in 5% milk and the following antibodies were used: PARP (cat no. 9532, Cell Signaling Technology), cleaved caspase 9 (cat no. 20750, Cell Signaling Technology), cleaved caspase 3 (cat no. 9664, Cell Signaling Technology), cytochrome C (cat no. 4272, Cell Signaling Technology), Bax (cat no. 41162, Cell Signaling Technology), ATF6 (cat no. 65880, Cell Signaling Technology), XBP1s (cat no. 27901, Cell Signaling Technology), PERK (cat no. 3192, Cell Signaling Technology), ATF4 (cat no. 11815, Cell Signaling Technology), CHOP (cat no. 2895, Cell Signaling Technology), actin (cat no. 3700, Cell Signaling Technology), and HRP-linked anti-mouse (cat no. 7076, Cell Signaling Technology) and anti-rabbit (cat no. 7074, Cell Signaling Technology) secondary antibodies.

### 2.5 Statistical analysis

Statistical analyses were performed using one-way ANOVA with Tukey’s multiple comparison test on GraphPad Prism 8. Data representations are plotted as mean ± SEM with significance levels set at *p<0.05, **p<0.01, ***p<0.001, ****p<0.0001.

## 3 Results

### 3.1 Screening assay standardization

Prior to the start of the screening procedure using the Screen-Well Natural Compound Library, a preliminary standardization of the screening assay used (Cell-Titer-Glo) was performed. Seeding density of the screening plates, along with the screening dose of natural product and the appropriate concentration of ER stressor tunicamycin, were established and applied throughout the entire screening process.

### 3.2 Initial screening of natural product library

The initial screen of the 502 compounds provided through the Enzo Screen-Well Natural Compound library was tested against the healthy corneal endothelial cell line 21T subjected to the ER stressor tunicamycin. The screening compounds were added as pretreatments 2 hours prior to incubation with tunicamycin, and cell viability was determined at 24 hours after addition of the ER stressor and screening compound (**Figure 1A**). Preliminary ‘hits’ were identified through the selection criteria of displaying a 30% or more increase in cell viability for 21T HCEnC treated with selected screening compounds and tunicamycin compared to 21T HCEnC treated with tunicamycin alone. With the threshold for selection set, we identified 74 compounds exhibiting 30% or more protection of 21T HCEnC cell viability in response to tunicamycin-induced stress (**Figure 1B**). Of the 74 identified compounds, one compound fell into the range of 50% protection or more, 14 showed cell viability protection in the range of 40-50%, while 59 compounds increased cell viability between 30-40% compared to tunicamycin-treated HCEnCs.

**Figure 1:**
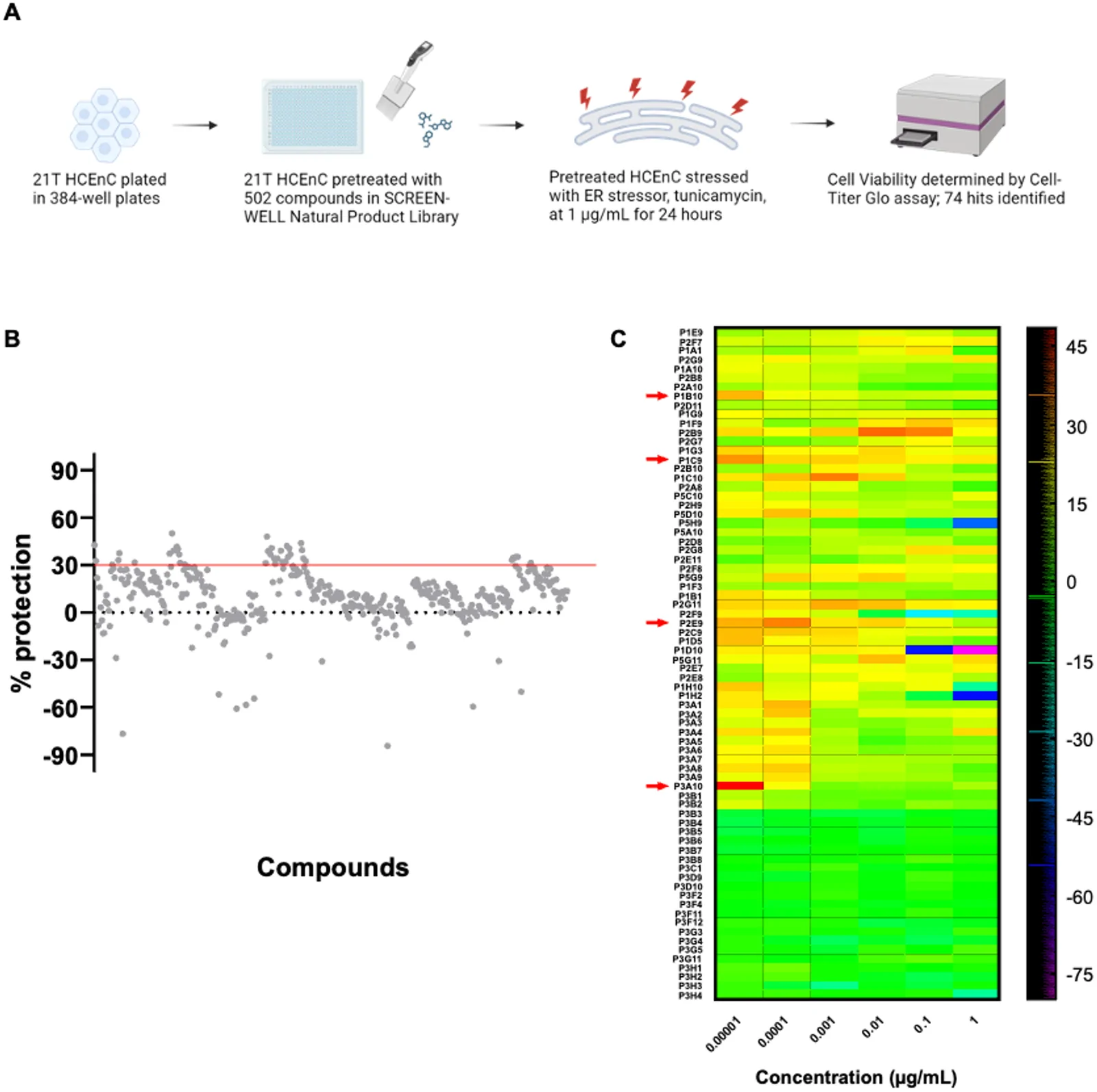
Successive rounds of screening of a natural product library in the healthy corneal endothelial cell line 21T and in the Fuchs corneal endothelial cell line F35T yielded 4 final compounds exhibiting rescue from ER stress. **(A)** Schematic flowchart of the preliminary screening protocol in 21T HCEnC. **(B)** Screening of the SCREEN-WELL ^®^ Natural Product library in 21T HCEnC yielded 74 preliminary hits exhibiting over 30% protection of cell viability. 21T HCEnC were pretreated with natural compounds at 0.01 µg/mL and co-incubated with tunicamycin 1 µg/mL for 24 hours. **(C)** Dose-response testing was applied to preliminary hits using untreated F35T HCEnC. The heatmap shown indicates the percentage increase in cell viability, identifying 4 hits with 30% protection or more in the lowest dose of natural compound tested (0.00001 µg/mL), denoted by arrows.

### 3.3 Dose-response screening of preliminary ‘hits’

The 74 ‘hits’ identified through the preliminary screen were then placed through a second round of screening, testing a range of 6 different doses from 10 pg/mL to 1 μg/mL. Untreated F35T cells were used to screen the preliminary hits, as the cells used are from a FECD patient-derived cell line containing 1500 CTG repeats in the *TCF4* allele and have been previously shown by our lab to express ER stress markers at a higher level at baseline compared to healthy 21T HCEnCs (8, 17). Selection for final hit compounds was determined by a similar selection criterion: an exhibition of cell viability improvement by 30% or more at the lowest dose of compound tested compared to viability of F35T cells at baseline. This secondary screen yielded 4 final compounds that provided protection of over 30% in viability (**Figure 1C**). The compounds are emodin, dubinidine, L-cycloserine, and cevadine. Cevadine, also known as veratrine, was selected for further analysis due to its function as a sodium channel agonist and its interaction with Na^+^/K^+^ ATPase channels, a functional ion transporter critical for maintaining corneal transparency that is downregulated in FECD (18–21).

### 3.4 Veratrine reduces mitochondrial-apoptotic markers in FECD cell line

To demonstrate changes in protein expression in response to treatment with the screening compound veratrine, we probed for apoptotic markers in F35T cells under tunicamycin-induced ER stress. We tested 4 doses of veratrine, with western blotting results for the most effective doses (0.001 μg/mL and 0.01 μg/mL) shown in **Figure 2**. Specifically, pretreatment with veratrine 0.01 μg/mL, along with tunicamycin, significantly reduced cleaved caspase 9, a mitochondria-induced initiator caspase, expression compared to F35T cells treated with tunicamycin only (**Figure 2B**). A similar trend in downregulation was seen in expression levels of cytochrome C, a mitochondrial protein whose release triggers the start of the intrinsic apoptotic pathway, and Bax, a proapoptotic protein that induces the release of cytochrome C, when tunicamycin-treated cells were co-incubated with veratrine compared to tunicamycin treatment alone (**Figure 2A**). Cleaved caspase 3, an executioner caspase, was also shown to be downregulated in ER-stressed F35T cells after treatment with veratrine compared to ER-stressed corneal endothelial cells alone (**Figure 2A**). Lastly, a significant decrease in poly(ADP-ribose) polymerase (PARP), a DNA damage sensor, expression levels was also observed post-veratrine treatment in F35T cells subjected to tunicamycin compared to tunicamycin treatment alone (**Figure 2C**). The other doses of veratrine (0.00001 μg/mL and 0.0001 μg/mL) did not significantly attenuate any mitochondrial-apoptotic markers in the veratrine-along-with-tunicamycin treatment group compared to tunicamycin alone (**Supplementary Figure 1A**).

**Figure 2:**
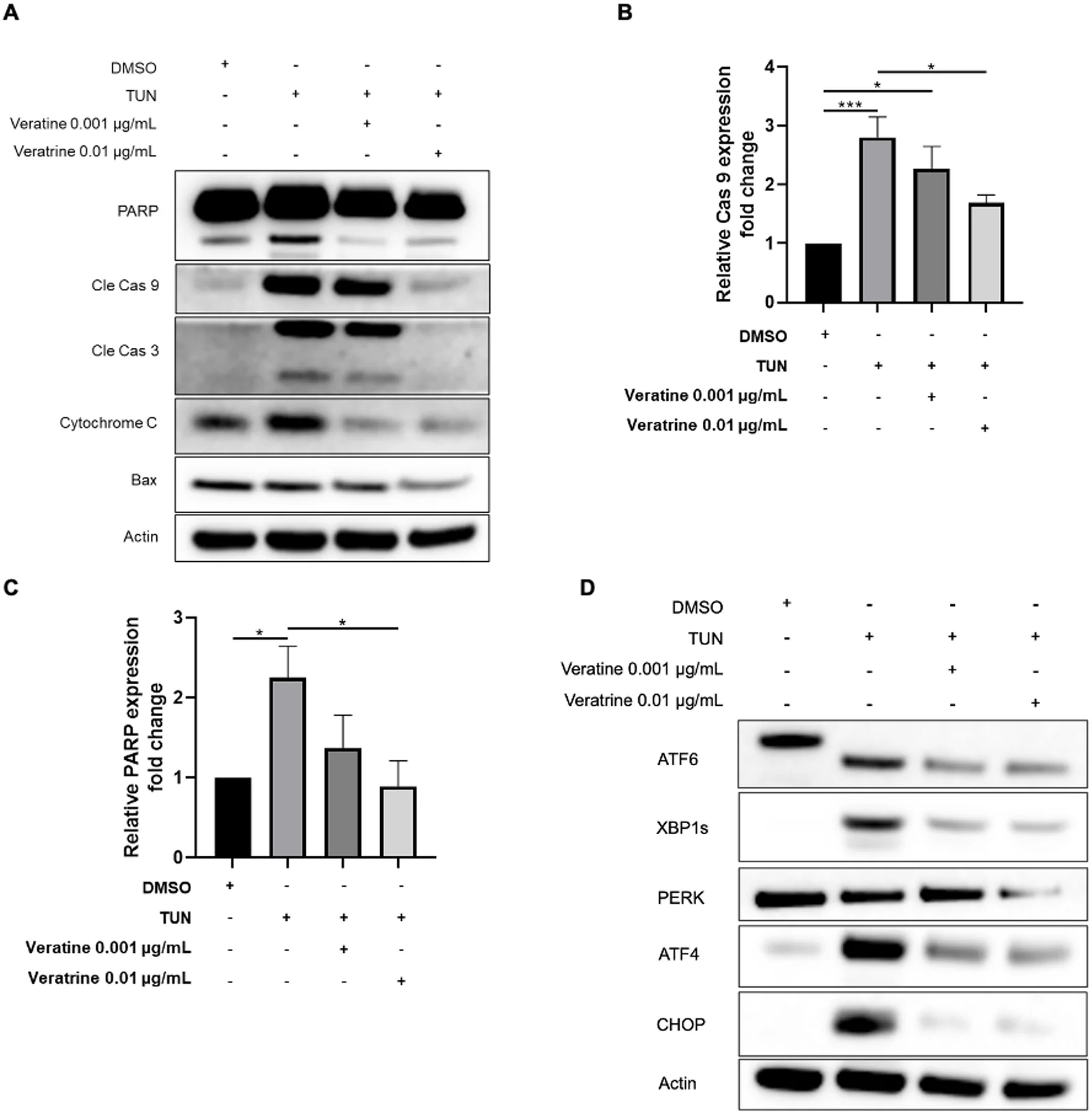
Application of veratrine to the FECD corneal endothelial cell line under ER stress reduced mitochondrial apoptotic markers and ER unfolded protein response markers. **(A)** Representative Western blots of the FECD cell line pretreated with veratrine (0.001 µg/mL or 0.01 µg/mL as indicated) then co-incubated with tunicamycin (1 µg/mL) for 24 hours, probing for DNA damage sensor (PARP), mitochondrial apoptotic markers (cleaved caspase 9, cytochrome C, Bax), and general apoptotic marker (cleaved caspase 3). **(B)** Quantification of relative fold change in expression of cleaved caspase 9, indicating a significant reduction of the mitochondrial-mediated apoptotic marker in tunicamycin-treated FECD cells after application of veratrine (0.01 µg/mL) compared to tunicamycin-treated cells alone (n=4, *p<0.05, ***p<0.001, one-way ANOVA with Tukey’s multiple comparison test). **(C)** Quantification of relative fold change seen in PARP expression, showing a similar significant reduction of the DNA damage sensor in veratrine-treated FECD cells under tunicamycin-induced ER stress compared to tunicamycin-treated FECD cells alone (n = 4, *p < 0.05, one-way ANOVA with Tukey’s multiple comparison test). **(D)** Representative Western blots of the FECD cell line under tunicamycin-induced ER stress, pretreated with veratrine (0.001 µg/mL or 0.01 µg/mL as indicated). Markers from all three ER UPR pathways were probed, including ATF6 (ATF6 pathway), XBP1s (IRE1-XBP1s pathway), and PERK/ATF4/CHOP (PERK-ATF4-CHOP pathway). Reduction of ER stress response signals was seen prominently at the higher dose of veratrine used (0.01 µg/mL) with a trend of stress signal reduction observed.

### 3.5 Alterations to expression of ER unfolded protein response markers post-veratrine treatment in F35T corneal endothelial cells

Following our observation of reduced apoptotic markers in cells pretreated with veratrine, we wanted to explore the effects of veratrine on the ER unfolded protein response (UPR) pathways, as ER stress contributes to apoptosis and degeneration in FECD (7). Western blotting revealed a similar trend of decreased expression across all three ER UPR pathways, marked by key proteins including ATF6, XBP1s (IRE1 pathway), and PERK-ATF4-CHOP (**Figure 2D**) in the veratrine-along-with-tunicamycin treatment group compared to tunicamycin alone. The other doses of veratrine (0.00001 μg/mL and 0.0001 μg/mL) did not attenuate any ER UPR markers (**Supplementary Figure 1B**).

## 4 Discussion

Natural products have previously been explored for their potential efficacy in ocular diseases, including glaucoma (22), diabetic retinopathy (23), and age-related macular degeneration (24). However, there are limited studies examining the role of natural compounds in reducing sustained ER stress for improving corneal endothelial cell (CEnCs) survival. Our study aimed to fill this gap by identifying natural compounds that can protect against ER stress in FECD.

Our screening of 502 compounds found in the SCREEN-WELL Natural Product library revealed 4 final ‘hits’ that provided an increase in cell viability in both the tunicamycin-treated normal corneal endothelial cell line as well as in the diseased F35T cell line. These four compounds were emodin, dubinidine, L-cycloserine, and cevadine (veratrine). Emodin is a naturally occurring anthraquinone compound that is a key active ingredient in the genus *Rheum* plants and a common component of Chinese herbal medicine. Emodin has been shown to suppress inflammation and fibrosis in both cultured subconjunctival fibroblasts and a mouse model of ocular alkali burn (25). Furthermore, emodin may have therapeutic potential in diabetic cataract, as it was found to inhibit ferroptosis through binding to p53 in an *in vitro* lens epithelial cell model, thereby attenuating glucose-induced damage to lens epithelial cells (26). The second compound our screening identified was dubinidine, a naturally occurring quinoline alkaloid extracted from plants of the Rutaceae family, with anticonvulsant properties (27). However, dubinidine has never been studied in ocular disease. The next compound identified through our screening was L-cycloserine, a cyclic analog of serine and alanine that inhibits various enzymes, including serine palmitoyl transferase. By inhibiting serine palmitoyl transferase, L-cycloserine can reduce levels of ceramide, a critical mediator of apoptosis of photoreceptor cells. These findings suggest potential therapeutic utility for L-cycloserine in retinal degenerative diseases by protecting photoreceptors from oxidative stress-induced apoptosis (28, 29). The final compound identified in our screen was cevadine, a major steroidal alkaloid component of veratrine, a naturally derived alkaloid mixture obtained from the seeds of the sabadilla plant. Veratrine acts as a voltage-gated sodium channel agonist, promoting sustained sodium influx into cells. In rat thalamic cells, Senatorov et al. (30) found that the veratrine-induced increase in intracellular sodium was associated with rapid upregulation of Na+/K+-ATPase, increasing pump density in membrane and cytoplasmic compartments by 39% and 54%, respectively (30). Although veratrine has not previously been studied in ocular disease models, its ability to enhance Na^+^/K^+^-ATPase expression prompted us to explore its potential utility in FECD, a condition characterized by markedly reduced endothelial pump density, caused by decreased Na^+^/K^+^-ATPase in advanced disease (31).

We selected veratrine for targeted analysis to further investigate its utility in FECD. Although 21T HCEnC cells were used for the screening phase of the study, our targeted experiments evaluating veratrine were conducted in diseased F35T cells to assess its effects in a diseased endothelial cell model, without the confounding of the rapid cell death observed in tunicamycin-treated 21T cells. We found that the higher dose of veratrine (0.01 μg/mL) significantly reduced markers of mitochondrial apoptosis, including cleaved caspase 9, cleaved caspase 3, cytochrome C, Bax, and poly(ADP-ribose) polymerase (PARP). Interestingly, veratridine, a component of veratrine, typically induces apoptosis through intracellular calcium overload, increased superoxide anion production, and DNA fragmentation (32). However, we found the opposite effect in this study. This is likely because veratrine, as a mixture of steroidal alkaloids with a greater concentration of cevadine relative to veratridine, has lower overall toxicity than purified veratridine.

Since we also found decreased activation of ER UPR pathways post-treatment with veratrine 0.01 μg/mL, we propose that the anti-apoptotic effect of veratrine occurs secondary to decreased ER stress. The mechanism by which veratrine attenuates ER stress remains unknown and should be a target of future studies. One possible explanation is that veratrine acts upstream of the UPR by attenuating oxidative stress. In fact, a recent study of another *Veratrum*-derived alkaloid found that veratramine mitigated oxidative stress, protecting pulmonary artery endothelial cells against particulate matter pulmonary injury (33). Another possible explanation is that veratrine stabilizes intracellular calcium levels, thereby decreasing ER stress. We hypothesized that since veratrine is a voltage-gated sodium channel agonist, sodium influx can lead to increased calcium entry through the Na^+^/Ca^2+^ exchanger. However, we did not find evidence of this mechanism in this study, as sodium-potassium pump protein expression in the FECD cell line did not change after exposure to the ER stressor tunicamycin or with pretreatment with veratrine (**Supplementary Figure 2**). Therefore, it is unclear whether these proposed mechanisms of veratrine would attenuate ER stress in corneal endothelial cells, thus requiring further investigation in future studies.

When considering the potential clinical utility of veratrine in FECD, it is essential to study its safety profile. Silva Freitas et al. (34) found that veratrine can lead to skeletal muscle toxicity, with a significant dose-dependent decrease in rat skeletal muscle mitochondrial function (34). This finding underscores the importance of determining veratrine’s therapeutic window to balance efficacy with safety. In our study, we found that the efficacy of veratrine in decreasing mitochondrial-apoptotic and ER UPR markers only occurred at the highest dose (**Figure 2**), with no effect at the two lower doses (**Supplementary Figure 1**), thus highlighting the potential difficulty of balancing veratrine’s therapeutic window with its safety profile in preclinical models in the future. Furthermore, veratrine’s recent registration as a pesticide in January 2025 (35) highlights its potency and further supports the need to evaluate its dose-dependent safety before its use in preclinical rodent models of FECD.

## 5 Conclusion

Our study found that of 502 natural compounds screened, veratrine may have a protective effect on ER-stressed CEnCs through reduction of ER stress and apoptotic markers. Future studies are needed to study its dose-dependent ocular safety and tolerability in pre-clinical models, clarify its mechanism of action in reducing ER stress and apoptosis of CEnCs, and determine its clinical efficacy in FECD.

## Data Availability

The original contributions presented in the study are included in the article/**Supplementary Material**. Further inquiries can be directed to the corresponding author/s.

## Supplementary material

Supplementary Figure 1 Detection of apoptotic markers and ER stress markers in veratrine-treated FECD corneal endothelial cells subjected to ER stress. Representative immunoblots of Fuchs’ corneal endothelial cell line (F35T) pretreated with veratrine (0.00001-0.0001 μg/mL) then subsequently incubated with ER stressor tunicamycin (1 μg/mL). **(A)** Elevation in expression levels of PARP (DNA damage sensor), cleaved caspase 9 and cytochrome C (mitochondrial apoptotic markers), as well as cleaved caspase 3 (general apoptotic marker), was detected in F35T treated with tunicamycin when compared to the control condition (DMSO). No differences in expression of PARP, Cle Cas 9, Cas 3 and Cytochrome C in the tunicamycin and veratrine group compared to tunicamycin alone. **(B)** Representative Western blots of the FECD cell line pretreated with veratrine (0.00001-0.0001 μg/mL) co-incubated with tunicamycin (1 μg/mL). Representative markers of the ER unfolded protein response pathways are shown to be increased, including ATF4/CHOP and XBP1s, while PERK protein levels are seen to be downregulated in tunicamycin compared to the DMSO group. No differences in the expression of PERK, ATF4, CHOP, and XBP1s were observed in the tunicamycin with veratrine group compared to tunicamycin treatment alone.

Supplementary Figure 2 Detection of functional membrane protein in pretreated Fuchs corneal endothelial cells post-tunicamycin treatment. Representative western blot of sodium-potassium pump protein (Na^+^K^+^ ATPase) expression in the FECD cell line after exposure to the ER stressor tunicamycin (1 μg/mL) following pretreatment with either **(A)** veratrine (0.00001-0.0001 μg/mL) or **(B)** veratrine (0.001-0.01 μg/mL).
